# *Toxoplasma gondii* Infection Regulates the Balance of Activating and Inhibitory Receptors on Decidual Natural Killer Cells

**DOI:** 10.1371/journal.pone.0055432

**Published:** 2013-02-05

**Authors:** Xiaoyan Xu, Mingdong Zhao, Xianbing Liu, Yuzhu Jiang, Haixia Zhang, Xiaoyu Zhai, Ling Zhang, Xuemei Hu

**Affiliations:** 1 Department of Immunology, Binzhou Medical University, Shandong, People’s Republic of China; 2 Department of Radiology, Yantai Affiliated Hospital of Binzhou Medical University, Shandong, People’s Republic of China; Charité, Campus Benjamin Franklin, Germany

## Abstract

Inhibitory receptors and activating receptor expressed on decidual natural killer (dNK) cells are generally believed to be important in abnormal pregnancy outcomes and induced adverse pregnancy. However, if *Toxoplasma gondii* (*T. gondii*) infection induced abnormal pregnancy was related to dNK cells changes is not clear. In this study, we used human dNK cells co-cultured with human extravillous cytotrophoblast (EVT) cells following YFP-*Toxoplasma gondii* (YFP-*T. gondii*) infection in vitro and established animal pregnant infection model. Levels of inhibitory receptors KIR2DL4 and ILT-2, their ligand HLA-G, and activating receptor NKG2D in human decidua, and NKG2A and its ligand Qa-1 and NKG2D in mice uterine were analyzed by real-time PCR and flow cytometry with levels of NKG2D significantly higher than those of KIR2DL4 and ILT-2 in vitro and in invo. The level of NKG2D was positively correlated with cytotoxic activity of dNK cells in vitro. Numbers of abnormal pregnancies were significantly greater in the infected group than in the control group. This result demonstrated that the increased NKG2D expression and imbalance between inhibitory receptors of dNK cells and HLA-G may contribute to abnormal pregnancy outcomes observed upon maternal infection with *T. gondii.*

## Introduction

*Toxoplasma gondii* is a widespread obligate intracellular protozoan parasite that can infect all warm-blooded animals including humans and mice [1]. *T. gondii* infection may result in maternal immune deregulation and can cause a variety of syndromes during pregnancy, particularly when infection occurs during the first trimester, such as miscarriage, spontaneous abortion, or fetal teratogenesis [2]. The mechanisms that cause these syndromes are not understood, although it is clear that a successful pregnancy involves subtle regulation of the immune system, which exerts a crucial influence on fetal development [3]. More than 70% of lymphocytes in the decidua in early pregnancy are natural killer cells. These cells are tolerant to fetal trophoblastic cells, even though NK cells are innate immune effectors, able to exert a prompt cytolytic activity on virally infected or cancerous cells without prior stimulation [4]. Activation of maternal NK cells can result in abnormal pregnancies, whereas suppression of NK cell activity may maintain the fetal allograft during pregnancy [5], [6]. Therefore, disturbance of maternal NK immune tolerance to the fetus may result in the high incidence of abnormal pregnancies observed in mothers infected with *T. gondii*.

Decidual NK (dNK) cells are in direct contact with invading trophoblasts and are considered to be important for fetal tolerance during pregnancy [7]. In humans, the interaction between dNK inhibitory receptors (KIR2DL4, ILT-2) and their ligand HLA-G on invading trophoblasts allows these fetal cells to evade dNK cells. In mice, a similar interaction between inhibitory receptor NKG2A/CD94 and its ligand Qa-1 plays an important role in protecting trophoblasts from lysis by uterine NK cells [8], [9]. These receptors can trigger lysis directly by cross-linking and are considered the major activation receptors involved in NK cytotoxicity [10], [11]. The function of NK cells is regulated by a balance between activating and inhibitory signals provided by their heterocladic receptor repertoire upon recognition of specific ligands expressed on invading trophoblast.

Recently, it was shown that the proportion of dNK cells with inhibitory receptors KIR2DL4 and ILT-2 decrease significantly during abortion [12]. Abnormalities of the NK activity are observed in most patients who spontaneously abort [13]. There have been no studies of the balance between inhibitory and activating signals on dNK cells or between inhibitory receptors and their ligands during abnormal pregnancies resulting from *T. gondii* infection. In this study, the expression of inhibitory receptors KIR2DL4 and ILT-2 and activating receptor NKG2D on human dNK cells and HLA-G expressed on human extravillous cytotrophoblast cells isolated from tissue taken early in pregnancy and then infected by *T. gondii* in vitro were measured by real-time PCR and flow cytometry. The same methods were used to measure levels of NKG2A, NKG2D, and Qa-1 in pregnant mice infected with *T. gondii*.

## Materials and Methods

### Sample Collection

Human dNK cells and human trophoblast cells were isolated from tissue taken from 36 patients undergoing voluntary abortions in the Department of Obstetrics and Gynecology, Chinese Medicine Hospital and Yuhuang Ding Hospital of Yan Tai. All voluntary abortions occurred during the first trimester, between 6 and 12 weeks of gestation. Decidual and villi tissues were rinsed in sterile saline solution, and decidual tissues, representing the maternal side of the fetal-maternal interface, were macroscopically separated from fetal tissue and placenta. The latter tissues were further handled according to the regulations at the Chinese Medicine Hospital. The decidual tissue was placed in α-MEM medium (Hyclone), supplemented with 12.5% fetal calf serum (FCS, Gibco), 12.5% horse serum, 100 IU/ml penicillin, and 100 IU/ml streptomycin (Sigma). The villi tissue was placed in DMEM-12 medium (Hyclone) supplemented with 10% FCS and 100 IU/ml streptomycin.

### Isolation and Culture of Human dNK Cells

**Figure 1:**
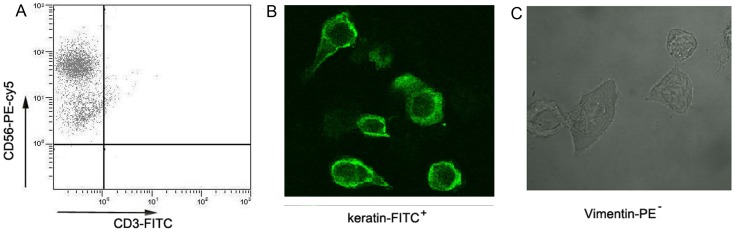
dNK cells and EVT cells identification. Phenotypic analysis of isolated CD3^−^CD56^+^ human dNK cells and human extravillous trophoblast cells using flow cytometry and immunohistochemistry. (A) dNK cells were isolated from fresh human deciduas tissues and were stained using anti-CD3-FITC antibody and anti-CD56-PE-cy5 antibodies, and then analyzed by flow cytometry. The CD3^−^CD56^+^ dNK cells were about 98% pure. Human extravillous trophoblast cells were isolated from fresh villi tissues and were stained with anti-keratin-FITC antibody and anti-vimentin-PE antibody and then detected by immunohistochemistry. (B) Trophoblast cells stained for keratin. (C) Unstained trophoblasts.

Decidual tissues were washed thoroughly with cold phosphate-buffered saline (PBS), minced with sterile scissors into 1- to 3-mm pieces, then digested with 0.1% collagenase type IV and 25 U/ml DNase I (Sigma-Aldrich) in α-MEM medium for 30 min at 37°C. The cell suspension was washed once with cold PBS, passed through a 75-µm pore-size nylon gauze and centrifuged at 1000 rpm for 5 min at room temperature. The cells were subjected to density gradient centrifugation on Ficoll-Hypaque Lymph (Sigma) at 2000 rpm for 20 min at 4°C to isolate the lymphocyte population. Lymphocytes were collected and washed with PBS twice and then centrifuged at 1500 rpm for 10 min at room temperature. The supernatant was discarded, and the dNK cells were purified by positive selection using CD56 antibody-coated magnetic MicroBeads (Miltenyi Biotech) and magnet-assisted cell separation. 30 samples were used to detect levels of KIR2DL4, ILT-2, and NKG2D; and 6 samples were used to do cytotoxicity assays. Each sample contained approximately 1.5×10^6^ (1.5×10^6^±3.2×10^4^) dNK cells. Approximately 7.5×10^5^ purified dNK cells co-cultured with human trophoblast cells were used as controls, and the rest were infected with *T. gondii*. Control and infected dNK cells were cultured in α-MEM medium supplemented with 12.5% FCS, 12.5% horse serum, 100 IU/ml penicillin, and 100 IU/ml streptomycin for 12 h at 37°C in a humidified 5% CO_2_ incubator. We first analyzed the protocol for purification of dNK cells from decidual tissue of uninfected pregnant women. A cell suspension containing about 95% CD3^−^CD56^+^ dNK cells from lymphocytes were obtained human NK Isolation Kit II (Miltenyi Biotech) (Fig. 1A).

### Isolation and Culture of Human Extravillous Cytotrophoblast Cells

The villi tissues were immediately washed with PBS and cut into 1- to 3-mm pieces. The villi tissues were digested with 0.25% trypsin (Sigma-Aldrich) and 0.02% DNase-I (Sigma-Aldrich) three times for 30 min each at 37°C with constant shaking. The dispersed trophoblast cells were filtered through a 75-µm pore-size nylon gauze and were loaded onto a discontinuous Percoll gradient of 25–65% (GE Healthcare), followed by centrifugation at 2000 rpm for 20 min to separate different cell types. These cells between the density markers of 1.049 g/ml and 1.062 g/ml were collected. Cells were washed twice with Hank’s solution, equilibrated at 37°C, and cultured for 1 hr in high-glucose, phenol-red-free DMEM (Hyclone) containing 20% FBS (Gibco), 2.5 mM L-glutamine, 15 mM HEPES, 100 U/mL penicillin, and 100 mg/ml streptomycin. The culture suspension was transferred to culture flasks previously coated with BD matrigel (1∶2, matrigel:DMEM). The cultures were kept at 37°C in 5% CO_2_ with saturating humidity. An immunohistochemistry method was used to identify extravillous cytotrophoblast (EVT) cells. Isolated human EVT cells were stained with FITC-anti- keratin antibody and PE-anti-vimentin antibody. EVT cells were keratin positive (Fig. 1B) and vimentin negative (Fig. 1C). This result indicates that we were able to isolate human EVT cells.

### Co-culture of Human dNK Cells with Human Extravillous Cytotrophoblast Cells and YFP- *T. gondii* Infection

YFP*-T. gondii* tachyzoites were a gift from Professor Striepen, the Center for Tropical & Emerging Global Diseases, University of Georgia, USA. YFP-*T. gondii* tachyzoites frozen in liquid nitrogen were quickly thawed in normal saline (NS) solution at 37°C, and then Kunming mice were inoculated with tachyzoites via the celiac route. Uncontaminated celiac suspensions diluted with PBS to 1×10^7^ tachyzoites/ml were inoculated into mice every 54–72 hours.

About 7.5×10^5^ human dNK cells were co-cultured with 7.5×10^5^ human EVT cells. After 12 h of co-culture, *T. gondii* tachyzoites at the concentration of 4.5×10^6^ were added to dNK cells at the ratio of 3∶1 (*T. gondii*: cells), and aliquots were removed following culture for 12 h, 24 h, 36 h, 48 h, and 60 h. Control cells were treated identically but were not infected with *T. gondii*. dNK and adherent EVT cells from both groups were centrifuged at 1000 rpm for 5 min and collected for real-time PCR and flow cytometry analysis.

### Animals

C57BL/6 mice (6- to 8-week-old females and 8- to 10-week-old males) were purchased from the Centre of Experimental Animals, Wei Tong Li Hua of Beijing. Mice were housed five per cage at 20–24°C on a 12 h light/12 h dark cycle, with unlimited supplies of food and water sterilized by irradiation and autoclaving. After overnight cohabitation with males at a ratio of 2∶1, females with a vaginal plug [gestational day (gd) 0] were segregated and randomized to experimental or control groups. All mouse experiments were performed according to national animal care guidelines, and all related data were approved for publication by the University’s Institutional Review Board.

### Infection and Pregnancy Outcomes

YFP-*T. gondii* tachyzoite RH strain was maintained by passage once every 54 hr in the peritoneal fluid of intraperitoneally (i.p.) infected mice. Pregnant mice were inoculated i.p. with 400 tachyzoites in 200 µl sterile PBS on gd 8, and the control animals were inoculated with 200 µl sterile PBS. The mice were sacrificed at 6 days post-infection (dpi), uteri and placenta were removed, and the total numbers of implantations and resorption sites (indicative of abortions) were counted. The resorption sites were identified by their small size and necrotic, hemorrhagic appearance compared with normal embryos and placenta. The percentage of abortions was calculated as the ratio of resorption sites to total implantation sites (resorptions plus normal implantation sites).

### Real-time Quantitative PCR

The two types of cells above were collected, stained with CD56-PE-cy5 antibody (eBioscience, USA) and HLA-G-PE (eBioscience, USA) antibody, and then isolated with fluorescence activated cell sorting. The CD56 positive cells were considered as human dNK cells and HLA-G positive cells were human trophoblast cells, repectively. Total RNA was extracted from human dNK cells co-cultured with human EVT cells and from mouse uteri and placenta and reverse transcribed into cDNA using random hexamer primers and RNase H minus reverse transcriptase (Fermentas) according to the manufacturer’s instructions. For real-time PCR, primers were designed to ensure specificity for the target mRNA and were synthesized by Sangon Biotech Co. Sequences are listed in Table 1. PCR reactions were performed using Sybr Green real-time PCR reagent (Fermentas) in a total volume of 20 µl. Real-time PCR conditions for human *KIR2DL4*, *ILT-2*, *NKG2D*, and mouse *NKG2A*, *Qa-1*, and *NKG2D* were 95°C for 1 min and then 40 cycles of 95°C for 15 s, 60°C for 15 s, 72°C for 45 s; the annealing temperature of HLA-G was 58°C. The CT values for samples were determined. Relative target gene mRNA expression was normalized to *β-actin* expression using theΔ^ct^ method. All reactions were carried out using a Corbett Rotorgene RG-3000 in triplicate.

**Table 1:** Sequences list.

Name	Sequences (5′-3′)	
KIR2DL4 (human)	Forward primer	CATGAACTTAGGCTCCCTGCA
	Reverse primer	CATGGAAAGAGCCGAAGCA
ILT-2 (human)	Forward primer	AGTGACGTATGCCGAGGTGAA
	Reverse primer	TCTTCCGCCTGTCTGTCCTTT
NKG2D (human)	Forward primer	ACCCAACCTACTAACAATAA
	Reverse primer	TACCGCTGGTGTAATCTC
HLA-G (human)	Forward primer	CTGACCCTGACCGAGACCTGG
	Reverse primer	GTCGCAGCCAATCATCCACTGGAG
Beta-actin (human)	Forward primer	TTGTTACAGGAAGTCCCTTGCC
	Reverse primer	ATGCTATCACCTCCCCTGTGTG
NKG2A (mouse)	Forward primer	GGATTACATCTCCCTGAACG
	Reverse primer	GGTATGCCCTCTGTTGGTG
NKG2D (mouse)	Forward primer	GGCTTGCCATTTTCAAAGAG
	Reverse primer	TGAGCCATAGACAGCACAGG
Qa-1 (mouse)	Forward primer	ACTGAAGGTGGCAAAGAAC
	Reverse primer	GCTGAAGTCCGAATAGATG
Beta-actin (mouse)	Forward primer	GAGCCTTCCTTCTTGGGTAT
	Reverse primer	TGGCATAGAGGTCTTTACGG

### Flow Cytometry

To analyze changes in receptor expression, the following fluorophore-conjugated murine monoclonal antibodies (mAb) were used: mouse-anti-human KIR2DL4-PE and anti-CD3-FITC (both from Biolegend), mouse-anti-human NKG2D-PE and PE-conjugated anti-mouse NKG2A monoclonal antibody and anti-human HLA-G-PE (all from eBioscience), and mouse-anti-human ILT-2-PE and PE-cy5-conjugated CD56 (both from BD Pharmingen), and PE-conjugated anti-mouse Qa-1 antibody (Santa Cruz Biotechnology). Samples from both in vitro experimental and control groups were incubated with antibodies at room temperature in the dark for 30 min and fixed with 4% paraformaldehyde. dNK cells were harvested, centrifuged at 1000 rpm for 5 min and stained with at room temperature for 30 min.

Single-cell suspensions were prepared from pregnant mouse placenta and uterine tissue by dissecting the tissue into small pieces, grinding, and then filtering through sterile nets. The erythrocytes were lysed before the samples were subjected to flow cytometry analysis. Uterine NK cells were stained with PE-conjugated anti-mouse NKG2A antibody and PE-conjugated anti-mouse Qa-1 antibody in the dark for 30 min at 4°C according to the manufacturer’s instructions to analyze CD94/NKG2A and Qa-1 expression. The antibody-conjugated cells from both groups were analyzed on a FACSCaliber (Becton Dickinson); data acquired was analyzed using CellQuest (Becton Dickinson).

### dNK Cytotoxicity Assays

To investigate the cytotoxic activity of dNK cells after infection with *T. gondii*, human EVT cells were used as target cells. Cytotoxicity assays were performed as previously described [reference]. Briefly, 2×10^4^ human extravillous trophoblast cells were incubated with the dye PKH67 (Sigma-Aldrich), which inserts into plasma membranes. Target cells were then incubated with 12 h, 24 h, 36 h, 48 h, and 60 h with infected or uninfected dNK cells. After the indicated time, cells were put on ice to terminate the reaction. A membrane-impermeable DNA stain, To-Pro-3 (Molecular Probes), was added to each culture (1 µM final concentration), and cells were analyzed by flow cytometry. PKH67 was excited at 49 0 nm and To-Pro-3 was excited at 633 nm using a He-Ne laser. Target cells were gated by PKH67 green fluorescence, whereas undamaged cells were distinguished from lysed cells by their ability to exclude the DNA stain. Background and maximum To-Pro-3 staining were determined by incubation in MEM medium and 1% NP-40 detergent. The percentage cytotoxicity was calculated using the following formula: Percentage cytotoxicity = Sample lysis - Spontaneous lysis/Maximum lysis - Spontaneous lysis × 100%.

### Ethics Statement

Sample collection procedures for this study were approved by University of Binzhou Medical College Ethics Committee, and informed written consent were obtained from all patients. The ethics committees approve this consent procedure. This study was carried out in strict accordance with the recommendations in the Guide for the Care and Use of Laboratory Animals of Binzhou Medical University. The protocol was approved by the Committee on the Ethics of Animal Experiments of Binzhou Medical University. All surgery was performed under sodium pentobarbital anesthesia, and all efforts were made to minimize suffering.

### Statistical Analysis

Data are presented as mean ± SEM. All data were processed with SPSS 13.0 statistical software. Paired t-tests were used to analyze differences in percentages of dNK subsets and levels of inhibitory receptors KIR2DL4 and ILT-2 and activating receptor NKG2D expression in human dNK cells and HLA-G expression in human EVT cells, and NKG2A, NKG2D and Qa-1 in mouse uterine cells between control groups and infection groups. Correlation analysis was used to analyze the correlations between receptor expression and dNK cytotoxicity. Two-tailed p values of less than 0.05 or 0.01 were considered significant or very significant, respectively.

## Results

### Expression of Human dNK Cells Receptor mRNA is Altered Following YFP-*T. gondii* Infection

**Figure 2:**
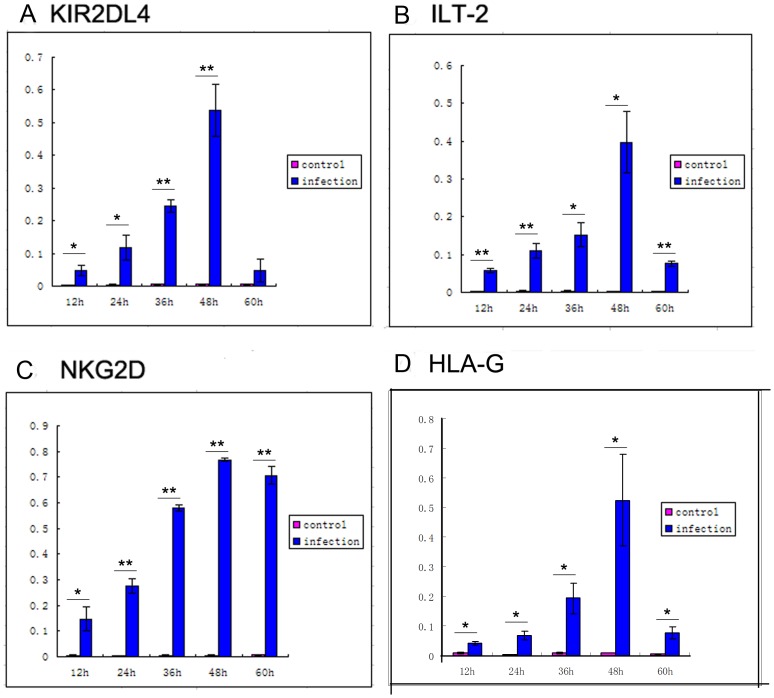
Levels of mRNA. The levels of (A) *KIR2DL4* and (B) *ILT-2*, (C) *NKG2D*, and (D) *HLA-G* mRNAs were measured by real-time PCR. Levels of all four receptors were significantly increased on infected cells compared to those on control cells at 12 h, 24 h, 36 h, 48 h, and 60 h. The magnitude of the increase in *NKG2D* expression was significantly higher than those of *KIR2DL4* and *ILT-2* (n = 30, *p<0.05, **p<0.01).

The levels of mRNAs encoding inhibitory receptors *KIR2DL4* and *ILT-2*, of activating receptor *NKG2D*, and of ligand *HLA-G* were measured by real-time PCR (Fig. 2). Levels mRNAs of all four receptors were significantly increased in infected cells compared to those in control cells at 12, 24, 36, 48, and 60 h post infection. The magnitude of the increase in *NKG2D* expression was significantly higher than those of *KIR2DL4* and *ILT-2* (n = 30, *p<0.05, **p<0.01).

### Human dNK Receptor Expression Alters After Co-culture with EVT Cells Following YFP-*T. gondii* Infection

The levels of inhibitory receptors *KIR2DL4* and *ILT-2*, and their ligand *HLA-G* and activating receptor *NKG2D* were measured by flow cytometry (Table 2). Levels of all receptors and ligand were significantly increased on infected cells compared to those on control cells at all time points. The magnitude of the increase in *NKG2D* expression was significantly higher than those of *KIR2DL4* and *ILT-2* (n = 30, *p*<0.05).

**Table 2:** Changes in levels of dNK receptors and HLA-G following *T. gondii* infection.^a^

		12 hr		24 hr		36 hr		48 hr		60 hr	
		C	I	C	I	C	I	C	I	C	I
KIR2DL4	x-mean	7.97	17.30^C^	8.57	26.53^C^	8.37	40.92^C^	8.90	55.17^C^	8.13	13.03
	sd	0.32	1.35	0.95	0.83	1.53	2.82	0.20	1.55	0.21	0.57
	p	0.0099		0.0017		0.0044		0.0004		0.0078	
ILT-2	x-mean	4.25	10.60^b^	5.50	23.10^b^	5.85	34.80^b^	4.85	41.10^b^	6.90	24.10
	sd	0.49	0.99	0.85	1.98	0.64	3.68	0.35	3.11	0.57	3.68
	p	0.0350		0.0290		0.0470		0.0340		0.0810	
NKG2D	x-mean	6.00	20.40^b^	6.50	30.70^C^	5.75	43.80^C^	7.00	64.30^b^	7.95	74.55^b^
	sd	0.14	0.99	0.99	1.27	1.63	1.41	0.42	1.98	0.35	4.03
	p	0.0350		0.0053		0.0025		0.0122		0.0296	
HLA-G	x-mean	4.25	10.60^b^	5.95	22.70^b^	5.95	30.85^b^	5.75	41.00^b^	5.86	22.95
	sd	0.49	0.99	0.36	1.56	0.78	1.34	0.78	1.56	1.06	2.40
	p	0.0390		0.0320		0.0380		0.0290		0.0910	

a Values are shown as means ± standard deviaions (10 samples per group).

b Significantly different from control group at p<0.05 based on paired t-test.

c Significantly different from control group at p<0.01 based on paired t-test.

### *T. gondii* Infection Leads to Abnormal Pregnancies in Mice

**Figure 3:**
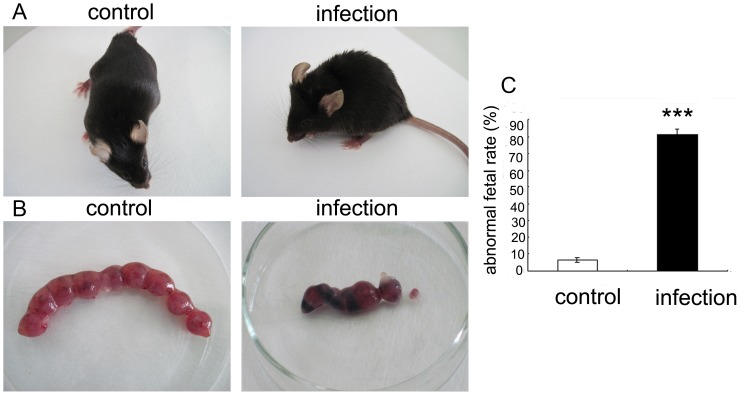
Pregnancy outcomes of mice following *T. gondii* infection. The impact of *T. gondii* infection on pregnant mice and their pregnancy outcome. (A) Infected mice exhibited mental and behavioral disorders and extrados, but control group appeared to be energetic. (B) Representative pictures of uteri from *T. gondii*-infected mice and gestational age-matched control mice 6 day post-infection (six dpi). (C) At six dpi, the abortion rate was calculated as the ratio of resorption sites to the total number of implantation sites in infected and control mice (***p<0.001). Data are expressed as the means ± S.E.M., and experimental and control group each contained six mice.

At six dpi, the pregnant mice infected with *T. gondii* showed malaise, extrados, and erected fur, whereas the control mice did not show abnormalities (Fig. 3A). As shown in Fig. 3B, in the infected mice but not control mice there was evidence of necrotic, hemorrhagic, and resorbed fetuses. The resorption rate for the infected group was significantly higher than that of control group (81.50±2.16%, n = 15 versus 6.56±1.29%, n = 15, P<0.001) (Fig. 3C). These results indicate that in our mouse model, the *T. gondii* infection results in abnormal pregnancy.

### Expression of dNK Cell Receptors and Ligands are Altered After Infection with YFP-*T. gondii*

**Figure 4:**
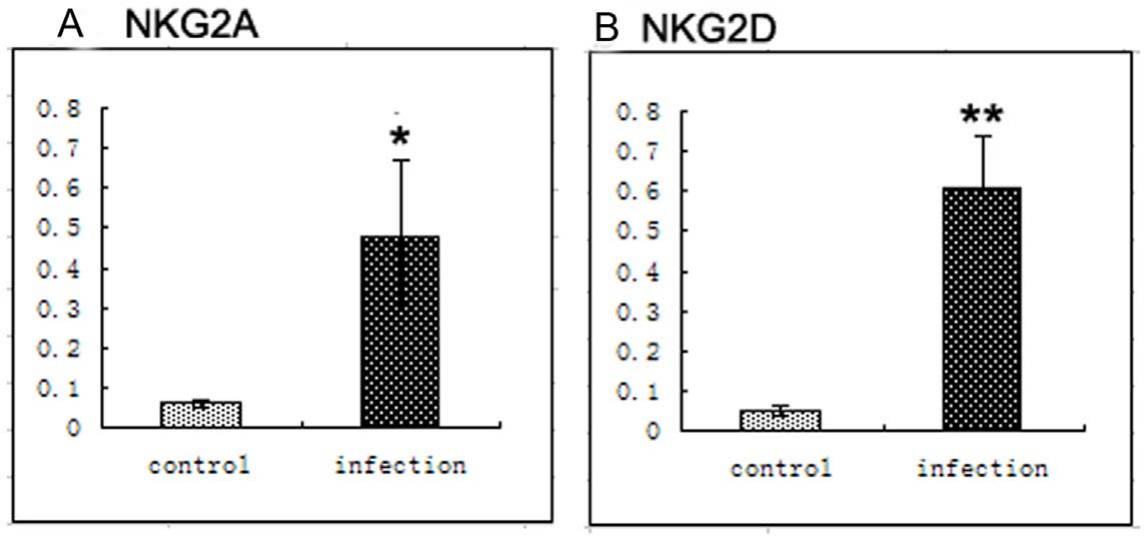
Levels of *NKG2A* and *NKG2D* mRNA expression in mice. After 12 h, 24 h, 36 h, 48 h, and 60 h of infection, the levels of (A) *NKG2A* and (B) *NKG2D* mRNA expression in uninfected and infected dNK cells were analyzed by real-time PCR (n = 15, *p<0.05, **p<0.01).

**Figure 5:**
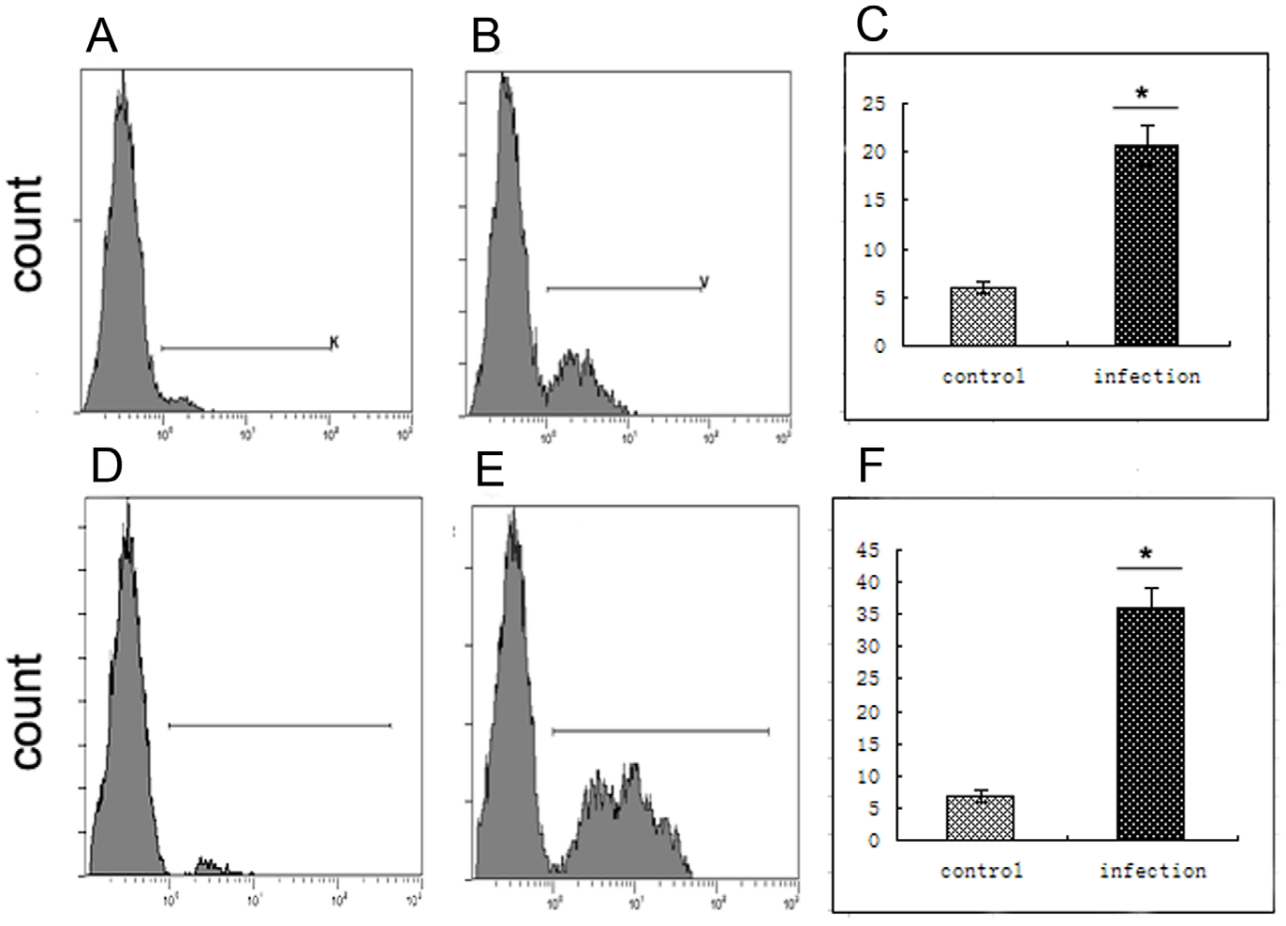
Levels of NKG2A and NKG2D in mice following *T.gondii* infection. Levels of NKG2A and NKG2D on dNK cells is altered by infection of mice with *T. gondii*. At 6 days post infection, the expression levels of NKG2A and NKG2D on the surface of uninfected (A, D) and infected (B, E) uterine and placental cells were analyzed by flow cytometry. (C, F) Data are presented as the means ± SEM (n = 15, *p<0.05).

Levels of *NKG2A* and *NKG2D* mRNAs were significantly increased on uterine NK cells from infected pregnant mice compared to those on control mice. The magnitude of the increase in *NKG2D* mRNA expression (Fig. 4B) was significantly higher than that of the increase in *NKG2A* mRNA (Fig. 4A) (n = 15, *p*<0.01). Protein levels of *NKG2A* (Fig. 5A–C) and *NKG2D* (Fig. 5D–F) were also increased on NK cells from infected mice compared to controls (n = 15, *p<0.01).

**Figure 6:**
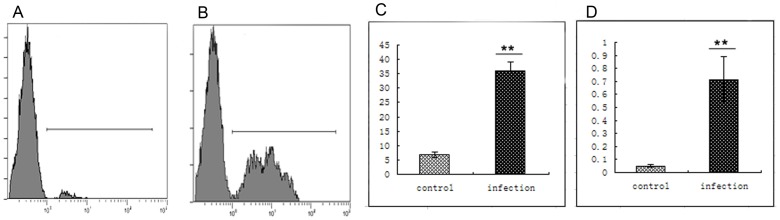
Levels of Qa-1 in mice following *T.gondii* infection. The levels of Qa-1 protein in placenta and uterus were determined by flow cytometry at six dpi. (A) Qa-1 protein levels in uninfected mice. (B) Qa-1 levels in infected mice. (C) (D) **P<0.01 versus control group. Experimental and control group each contained six mice.

We also used flow cytometry and real-time PCR to investigate the level of Qa-1 on trophoblasts from pregnant mice. As shown in Fig. 6A–C, the proportion of Qa-1 was increased during in pregnant mice infected with *T. gondii* relative to controls. The *Qa-1* mRNA levels were also increased due to infection (Fig. 6D) (n = 15, **P<0.01).

### Correlation between dNK Cytotoxic Activity and Receptor Expression

**Figure 7:**
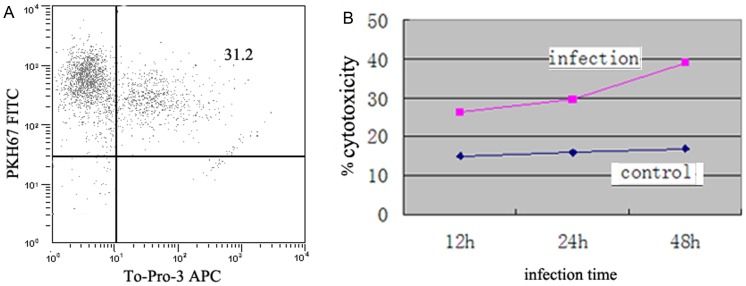
dNK cytotoxicity assays. The cytotoxicity caused by *T. gondii* was measure by co-staining uninfected and infected dNK cells with PKH67 and To-Pro-3 followed by flow cytometry analysis. (A) Total human extravillous trophoblast cells were stained with PKH67. Lysed cells are observed in the upper right frame. (B) Percentages of lysed human extravillous trophoblast cells in the presence of dNK cells incubated for 12 h, 24 h, or 48 h with *T. gondii*. Cytotoxicity of uninfected dNK cells was also determined. Data shown are averages of three independent experiments.

To investigate the cytotoxic activity of dNK cells after infection with *T. gondii*, human extrovillous trophoblast cells were used as target cells. A double-staining flow cytometry protocol was used to distinguish lysed extravillous trophoblast cells from undamaged cells (Fig. 7A). The cytotoxic activity of dNK cells infected with YFP-*T. gondii* increased with the time after infection and was significantly higher than that of the uninfected cells (Fig. 7B). Approximately 30% of target cells were lysed when incubated with dNK cells that had been incubated with virus for 48 h; only about 16% of target cells were dead when incubated with control cells (*p* = 0.0037). Percentages of lysed cells in these two groups at 12 and 24 h were 26.42±8.64% vs. 15.97±9.32% and 29.86±9.65% vs. 17.72±8.38%, respectively (*p* valves of 0.0125 and 0.0108, respectively). There were clear correlations between the cytotoxic activity and the protein levels of NKG2D (r^2^ = 0.759, *p*<0.05).

## Discussion

*T. gondii* is one of the five most prevalent pathogens responsible for chronic non-bacterial perinatal infections; known as TORCH pathogens, these agents can cause fetal teratogenesis during pregnancy in humans and in mice [14]. A large population of natural killer cells is a distinctive feature of the uterine mucosa during pregnancy [15], [16]. A method was successfully established in our lab to purify and identify the dNK cell subsets [17]. Using this method, we purified dNK cells and found that both dNK and EVT cells could be infected by *T. gondii*. dNK cells were more readily infected with *T. gondii* than were EVT cells, suggesting that *T. gondii* may invade and replicate in dNK cells.

NK cell functions are tightly regulated by a balance between activating and inhibitory signals, transduced by distinct receptor types [18]. KIR2DL4 and ILT-2 are important inhibitory receptors in humans and NKG2A is the major inhibitory receptor in mice; these receptors recognize HLA-G and Qa-1 expressed on human extravillous cytotrophoblast and mice trophoblast cells, respectively [19], [20]. These receptor-ligand interactions are related to the control of normal trophoblast invasion and protection of fetal trophoblasts from lysis by dNK cells. The activating receptor, NKG2D, is also expressed on dNK cells and its ligand is expressed on trophoblasts to control trophoblast invasion [21], [22]. If these interactions fail to function properly, abnormal pregnancy outcomes may result. The expression of NKG2D at the fetal–maternal interface is increased in most patients who spontaneously abort [23], [24]. In our study, we found that *T. gondii* infection upregulated the expression of NKG2D in human and in mice. This increase might trigger a higher cytotoxicity of dNK cells toward fetal cells. Interestingly, we also found that the magnitude of the increase in expression of activating receptor NKG2D in human and NKG2A in mice were higher than increases in levels of inhibitory receptors after *T. gondii* infection. Such changes may disrupt the balance between activating and inhibitory signals. In addition, levels of ligands HLA-G in human EVT cells and Qa-1 in mice were also increased, a further indication that inhibitory signals were disrupted. When activating signals prevail over inhibitory signals, an abnormal degree of dNK killing activity may result in pregnancy failure.

In addition, the cytotoxicity of dNKs toward human extravillous trophoblast cells was enhanced by *T. gondii* infection. The degree of cytotoxicity correlated positively with the levels of NKG2D relative to KIR2DL4 and ILT-2 and positively with level of NKG2D. In mice, the fetal resorption rate was significantly increased in infected mice compared to control mice in agreement with our previous findings [25], [26]. The results reported here provide additional evidence to suggest that the imbalance between inhibitory receptor and activating receptor enhances cytotoxicity of dNK cells.

### Conclusions

Our data reveal a link between *T. gondii* infection of dNK cells and abnormal pregnancy outcomes. Imbalances in the signals that regulate cytotoxic likely lead to the failure of pregnancy or abnormal fetal development in mothers infected with *T. gondii* early in pregnancy. Our results thus shed light on the molecular immune mechanism underlying abnormal pregnancy caused by *T. gondii* infection in human and in mice.
